# Supplementary material for: Genome-wide identification and expression analyses of SWEET gene family reveal potential roles in plant development, fruit ripening and abiotic stress responses in cranberry (Vaccinium macrocarpon Ait)
Source: PeerJ. 2024 Sep 19;12:e17974. doi: 10.7717/peerj.17974 (PMC11416763; doi:10.7717/peerj.17974)
Supplement: Supplemental Information 6 [file peerj-12-17974-s006.docx]

**MIQE checklist**

**1 Experimental design**

**1.1 Definition of experimental and control groups**

Table 1 The expression of *VmSWEET* genes in cranberry tissues and fruits at different development stages

| **Sampling name** | **Sampling period** | **Sampling name** | **Sampling period** |
| --- | --- | --- | --- |
| roots | flowering phase | Fruits at young stage (S1) | 10 days after full bloom |
| upright stems | flowering phase | Fruits at expansion stage (S2) | 30 days after full bloom |
| leaves of upright stem | flowering phase | Fruits at color turning stage (S3) | 60 days after full bloom |
| runner stems | flowering phase | Fruits at maturity stage (S4) | 80 days after full bloom |
| leaves of runner stem | flowering phase |  |  |
| flowers | flowering phase |  |  |

Table 2 Expression pattern under of *VmSWEET* genes underdifferent abiotic stresses

| **Drought treatment (20% PEG 8000)** | **Sampling period** | **saline-alkaline treatment**  **(30 mM Na_2_CO_3_ and 30 mM NaHCO_3_)** | **Sampling period** |
| --- | --- | --- | --- |
| CK | 0 h | CK | 0 h |
| T1 | 3h | T1 | 3h |
| T2 | 6h | T2 | 6h |
| T3 | 9h | T3 | 9h |
| T4 | 12h | T4 | 12h |
| T5 | 24h | T5 | 24h |
| **saline treatment (200 mM NaCl)** | **Sampling period** | **aluminum treatment (5mM AlCl_3_)** | **Sampling period** |
| CK | 0 h | CK | 0 h |
| T1 | 3h | T1 | 3h |
| T2 | 6h | T2 | 6h |
| T3 | 9h | T3 | 9h |
| T4 | 12h | T4 | 12h |
| T5 | 24h | T5 | 24h |

**1.2 Number within each group**

Cranberry tissues and fruits at different development stages were collected from 3 plants. Tissue cultured seedling were irrigated with different concentrations to to simulate abiotic stress, 5 bottles per gradient treatment.

**2 Sample**

**2.1 Description**

The cultivar Bain 11, in the small berry germplasm resource garden of Jilin Agricultural University (43°48′05′′N, 125°24′15′′E), was used to detect the expression of *VmSWEET* genes in cranberry tissues and fruits at different stages of development (Figure 1). The average annual precipitation in this area is 600–700 mm, with an average temperature of 4.6 ℃ and an annual frost-free period lasting 140–150 d. In order to improve the garden soil for optimal cranberry growth, it was mixed with sand, peatmoss, perlite, and sulfur powder. The pH of the improved soil was 5.0, which is conducive to the successful cultivation of cranberries. Roots, upright stems, leaves of upright stem, runner stems, leaves of runner stem, and flowers were collected during flowering period. Fruits at young stage (S1), expansion stage (S2), color turning stage (S3), and maturity stage (S4) were collected 10, 30, 60, and 80 d after full bloom, respectively. Tissue and fruit samples were haphazardly obtained from three trees and replicated three times.

Tissue-cultured seedlings of Bain11 were used to detect expression patterns under abiotic stress. An osmotic treatment (20% PEG 8000), saline treatment (200 mM NaCl), saline-alkaline treatment (30 mM Na_2_CO_3_ and 30 mM NaHCO_3_), and aluminum treatment (5mM AlCl_3_) were administered by immersing the roots of plantlets in containers with different solutions. Tender stem apexes were collected 0, 3, 6, 9, 12, and 24 h during the different stress treatments. Samples were collected from three containers every time and replicated thrice. All samples were immediately frozen in liquid nitrogen and stored at -80 °C.

**2.2 If frozen, how and how quickly?**

All fresh plant samples were frozen in liquid nitrogen immediately and then stored at -80 °C.

**3 Nucleic acid extraction**

**3.1 Procedure**

1. Warm extraction buffer（2%CTAB, 2% PVP, 100 mM Tris- HCl (pH 8.0), 25 mM EDTA, 2.0 M NaCl, 3.45mM spermidine）to 65°C in a heat block.
2. Grind samples to a fine powder in liquid nitrogen with a precooled pestle and mortar. Weigh 1–2 g of the powder into 8 sterile 2 mL Eppendorf tubes. Keep the tubes on ice until the next step.
3. Add 750 µL preheated extraction buffer to each sample and mix completely by inverting the tube. Incubate the tubes for 10 min in 65°C. When the materials are ripe fruits and roots, incubate timewas extended to 20-30 min.Within the incubation time vortex each sample or homogenize with homogenizator for a few seconds. Centrifuge the tubes at 12000 rpm for 10 min at 4°C.
4. Remove the supernatants to new Eppendorf tubes and extract twice with an equal volume of chloroform:IAA(Chloroform:isoamyl:alcohol=24:1), separating the phases at 12000 rpm at4°C.
5. Add 1/4 vol 10 M LiCl to the supernatant and mix gently. The RNA is precipitated overnight (on ice) at 4°C.
6. Centrifuge the tubes at 12000 rpm for 20 min at 4°C.
7. Decant the supernatant and press the tubes against a paper towel for a moment.
8. Wash the pellet with 1000 µL 70% ice-cold ethanol. Centrifuge the tubes briefly and decant the ethanol. For ripe fruits repeat the washing step at least twice, or until the dark color has disappeared.
9. Dissolve the RNA pellets in 100 µL of SSTE buffer(1.0 M NaCl, 0.5% SDS 10 mM Tris-HCl (pH 8.0), 1mM EDTA (pH 8.0)). If the samples dissolve slowly, the tubes may be heated briefly at 65°C.Merge 8 tubes to 2 tubes or 1 tube.
10. Extract the contents of the tubes once with an equal volume of phenol:chloroform:IAA (25:24:1) and once with equal volume of chloroform:IAA.
11. Add two volumes of ice-cold absolute ethanol to the supernatant, precipitate at –20°C for 2 h or at –70°C for 30 min.
12. Centrifuge the tubes at 12000 rpm for 20 min at 4ºC. Wash the pellet with ice-cold 70% ethanol and dry it in a speed vac for 30 min. Resuspend the pellet in DEPC-treated water.

**3.2 Purity and concentration of RNA**

The Purity and concentration of RNA were assessed by NanoPhotometer® spectrophotometer (IMPLEN P330).

| Sample Name | concentration（ng/μL） | A260/A280 | A260/A230 |
| --- | --- | --- | --- |
| S1 | 778 | 2.091 | 2.416 |
| S2 | 534 | 1.991 | 2.32 |
| S3 | 600 | 1.985 | 2.076 |
| S4 | 364 | 2.193 | 2.364 |
| Roots | 266 | 1.985 | 2.293 |
| upright stems | 1630 | 2.079 | 2.258 |
| runner stems | 1286 | 2.054 | 2.264 |
| leaves of upright stem | 1018 | 2.121 | 2.283 |
| leaves of runner stem | 822 | 1.995 | 2.461 |
| flowers | 292 | 2.086 | 2.281 |
| Saline treatment (0h) | 308 | 2.019 | 2.3216 |
| Saline treatment (3h) | 296 | 1.958 | 2.239 |
| Saline treatment (6h) | 152 | 1.944 | 2.414 |
| Saline treatment (9h) | 186 | 2.189 | 2.290 |
| Saline treatment (12h) | 191 | 2.058 | 2.341 |
| Saline treatment (24h) | 164 | 1.975 | 1.997 |
| Saline-alkaline treatment (0h) | 152 | 1.944 | 2.414 |
| Saline-alkaline treatment (3h) | 144 | 1.900 | 1.973 |
| Saline-alkaline treatment (6h) | 322 | 1.959 | 2.436 |
| Saline-alkaline treatment (9h) | 156 | 1.949 | 2.442 |
| Saline-alkaline treatment (12h) | 182 | 1.998 | 2.433 |
| Saline-alkaline treatment (24h) | 142 | 2.044 | 2.314 |
| Aluminum treatment (0h) | 336 | 2.093 | 2.246 |
| Aluminum treatment (3h) | 458 | 2.091 | 2.361 |
| Aluminum treatment (6h) | 213 | 1.978 | 2.230 |
| Aluminum treatment (9h) | 180 | 2.092 | 2.348 |
| Aluminum treatment (12h) | 152 | 2.158 | 2.297 |
| Aluminum treatment (24h) | 140 | 2.000 | 2.333 |
| Drought Streatment (0h) | 237 | 2.106 | 2.324 |
| Drought treatment (3h) | 304 | 2.008 | 2.254 |
| Drought treatment (6h) | 253 | 1.987 | 2.300 |
| Drought treatment (9h) | 177 | 2.086 | 2.298 |
| Drought treatment (12h) | 152 | 1.944 | 2.414 |
| Drought treatment (24h) | 156 | 2.015 | 2.273 |

**3.3 RNA integrity: method**

The integrity of RNA were assessed using electrophoresis on 1.2% agarose gels.

M_1_  1 2 3 4 5 6 7 8


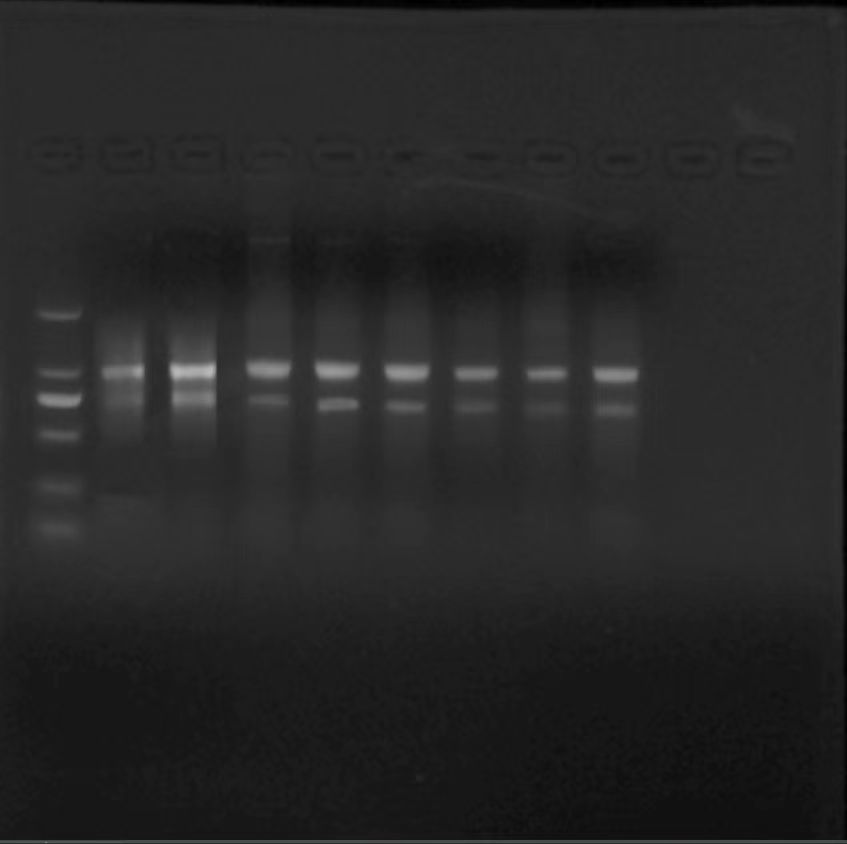


Fig. 1 1.2% agarose gel electrophoresis detection of total RNA extracted from cranberrytissues and fruits at different development stages

(M_1_: 20000 bp DNA Marker; 1~8: Random selection of total RNA from tested cranberry samples)

M_2_ 1 2 3 4 5 6 7 8 9 10 11 12 13 14 15 16 17


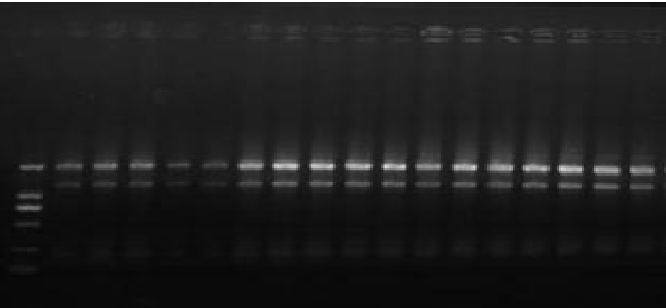


Fig. 2 1.2% agarose gel electrophoresis detection of total RNA extracted from cranberryleaf underdifferent abiotic stresses

(M_1_: 10000 bp DNA Marker; 1~17: Random selection of total RNA from tested cranberry samples)

**4 Reverse transcription**

**4.1 Complete reaction conditions**

Extracted RNA was reverse transcribed into cDNA using a *TransScript*® Uni One-Step gDNA Removal and cDNA Synthesis SuperMix (TransGen Biotechnology, AU311-02).Operation steps refer to the manual.

**4.2 Amount of RNA and reaction volume**

Amount of RNA: 1 μg; reaction volume: 20µL.

**4.3 Priming oligonucleotide (if using GSP) and concentration**

Anchored oligo (dT)_20_ (0.5µg/µL)

**4.4 Reverse transcriptase and concentration**

*TransScript*® Uni RT/RI enzyme mix.

**4.5 Temperature and time**

Incubate at 50°C for 15min to synthesize the firststrand of cDNA, 85 ℃ for 15 min to inactivate *TransScript*® Uni RT/RI and gDNA Remover.

**5 qPCR target information and qPCR oligonucleotides**

| Gene symbol | Sequence accession number | Forward primer (5'->3') | Reverse primer (5'->3') | Amplicon length |
| --- | --- | --- | --- | --- |
| *VmSAND* | CL5626.Contig_All | ATGTTGTCTTCTCTTCTCTCGTCC | AAAGAGGACACCAGAATCAGCTAC | 97 |
| *VmSWEET10.1* | vmacro16733 | TCGCTGATGATTGTCGGCTT | AGAGGTGCTGCAAATACGCT | 128 |
| *VmSWEET10.2* | vmacro19147 | GTGTTTCTTTCTCCGCTGCC | GCACCATCGCACTGAACAAG | 106 |
| *VmSWEET12* | vmacro19148 | TCAATGCTGTTGCGTGGTTC | CGTTTCGCCACCGTTGATTT | 257 |
| *VmSWEET1.1* | vmacro00890 | TGCTCTCTGCTTGGTATGGC | GACCACAAACGTGACAAGCC | 167 |
| *VmSWEET16* | vmacro08173 | GTGGTGACGACGAAGAGTGT | CCGCAAGGATAACATCGTGC | 115 |
| *VmSWEET1.2* | vmacro05470 | TTGCTGCCTCCATATTCTCCA | ATGGCATGTACTCCACGCTC | 93 |
| *VmSWEET3* | vmacro06571 | GCAGTTGGAGTGATGGGGAA | ACAACAGGCAAGCCATACCA | 167 |
| *VmSWEET2.1* | vmacro09417 | GATGTTTGGGTTGCTGCTGG | GTGTCGAACAGTGAAGTCGC | 88 |
| *VmSWEET13* | vmacro16734 | GTCTACCTTGCTCCAGTGCC | GGGTCACGGTCAACATCCTC | 244 |
| *VmSWEET4* | vmacro18238 | TCCCCATTCCCAACGGTCTA | TGACGGTAATGTTGCCAGGG | 139 |
| *VmSWEET14* | vmacro01036 | TGCTTCAGGTTGTCCGTACC | AAACCCACTACGTTCGGGAC | 142 |
| *VmSWEET5* | vmacro19373 | TGCATACCACCAAGCGAAGA | ACAAGGCCGTTCAGGAAGTT | 184 |
| *VmSWEET2.2* | vmacro03987 | GCTGGAATTGCTGGGAACCT | GGCAACAGGTATAACGTCCG | 189 |

**6 qPCR protocol**

**6.1 Complete reaction conditions**

qRT-PCR was performed on ABI StepOne Plus Real-Time Quantitative PCR System (Applied Biosystems, Foster City, CA, USA) using *PerfectStar*t® Green qPCR SuperMix (TransGen Biotechnology).

**6.2 kit identity and manufacturer**

*PerfectStar*t® Green qPCR SuperMix (TransGen Biotechnology, AQ601)

**6.3 Additives**

*2*×*PerfectStart*® Green qPCR SuperMix(TransGen Biotechnology, AQ601-01)

**6.3 Reaction volume and amount of cDNA/DNA**

| **component** | **volume** | **Final concentration** |
| --- | --- | --- |
| template | Variable | As required |
| Forward primer(10µM) | 0.4µL | 0.2µM |
| Reverse primer(10µM) | 0.4µL | 0.2µM |
| 2×PerfectStart® Green qPCR SuperMix(optional) | 10µL | 2× |
| Passive reference dye | Variable | 2× |
| Nuclease-free water | 0.4µL | - |
| Total | 20µL | - |

**6.4 Complete thermocycling parameters**

95 ◦C30 s;

95 ◦C5 s;

40-45cycles

60 ◦C30 s;

Dissociation stage

**7 qPCR validation**

**7.1 Specificity (gel, sequence, melt, or digest)**

*VmSAND*
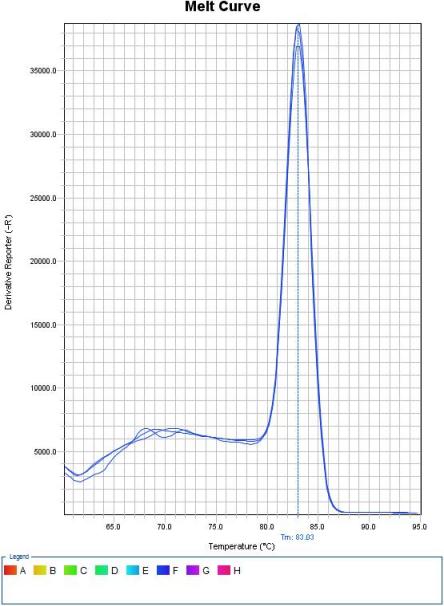

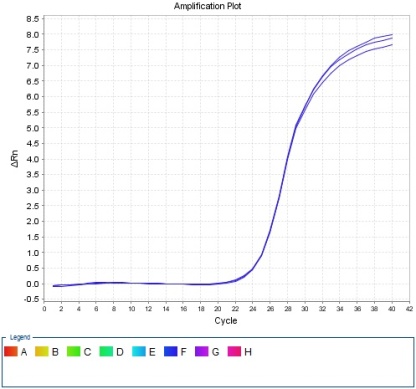


*VmSWEET10.1*
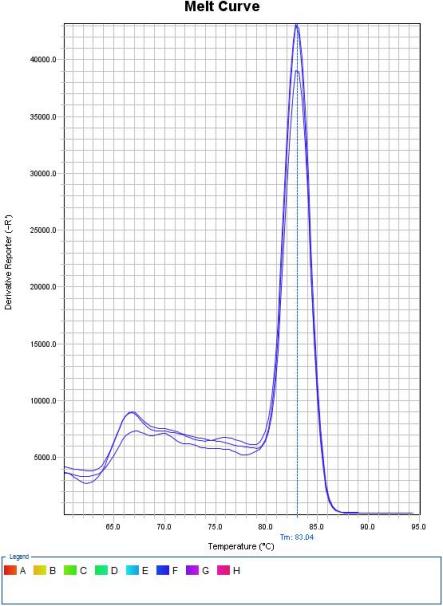

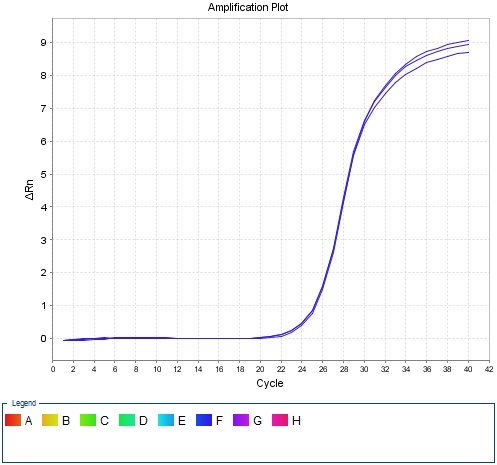


*VmSWEET10.2*
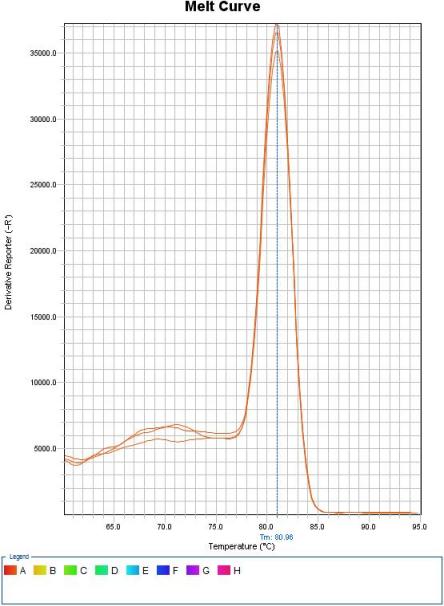

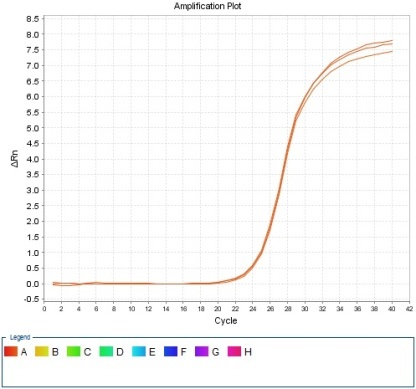


*VmSWEET12
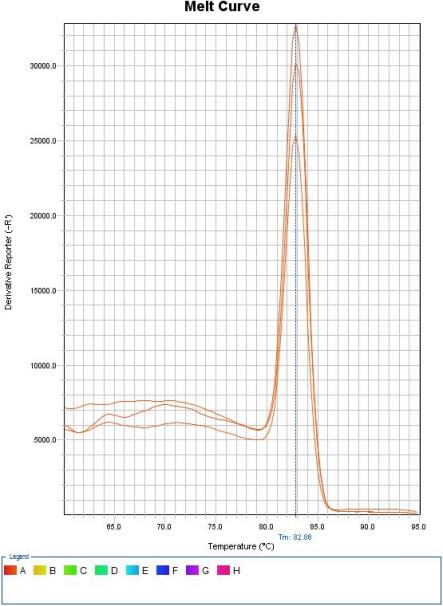
*
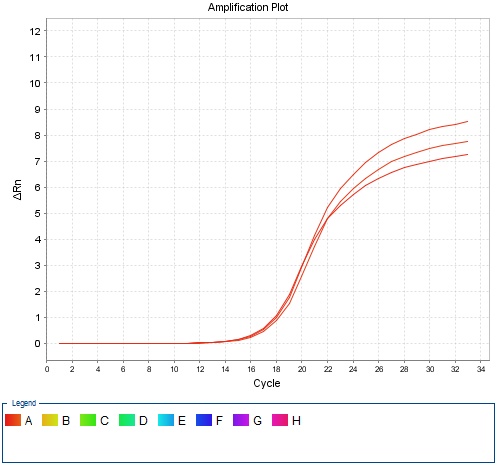


*VmSWEET1.1*
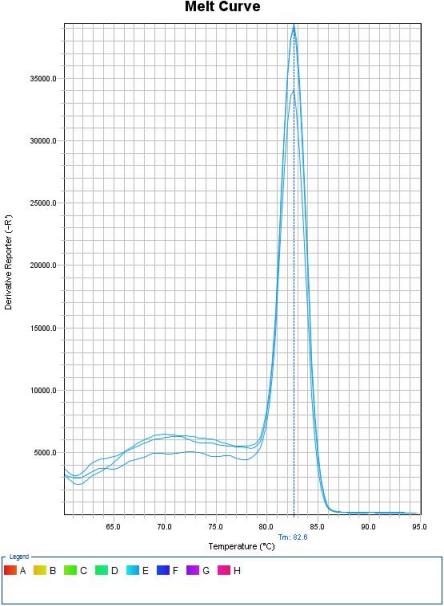

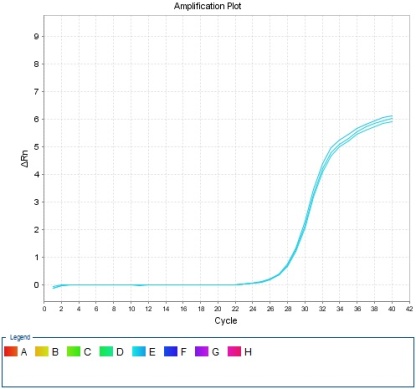


*VmSWEET16*
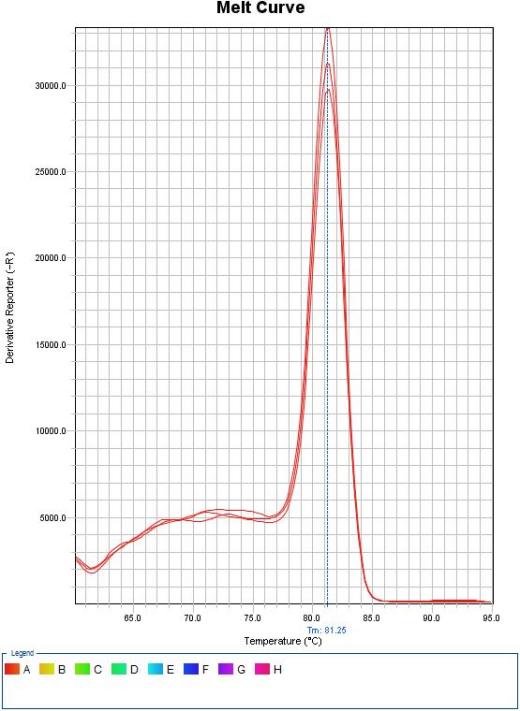

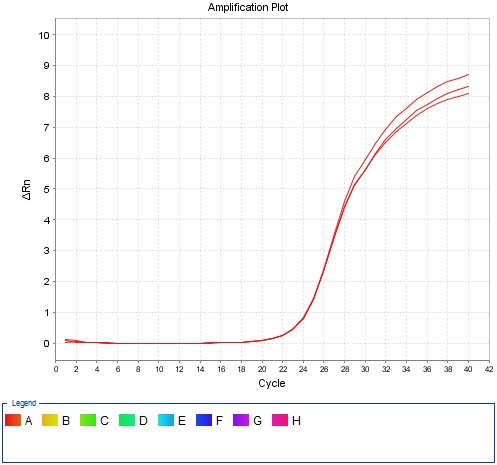


*VmSWEET1.2*
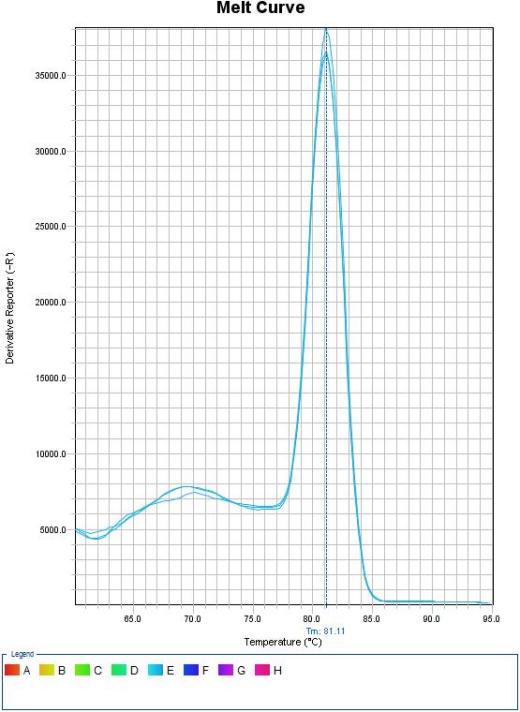

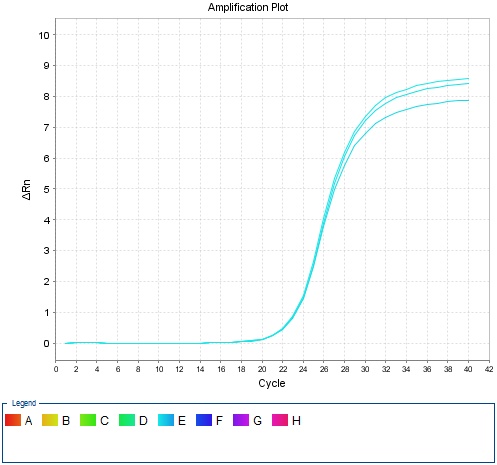


*VmSWEET3*
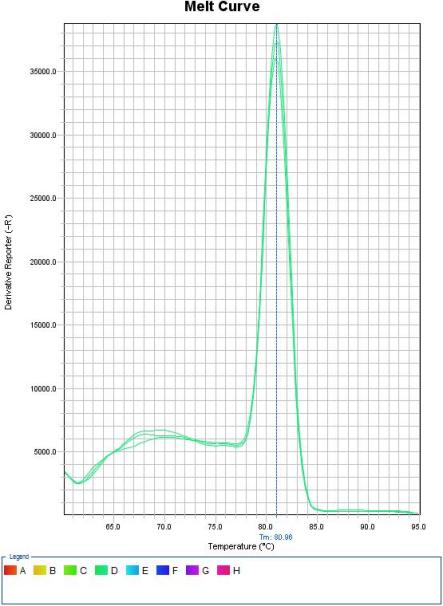

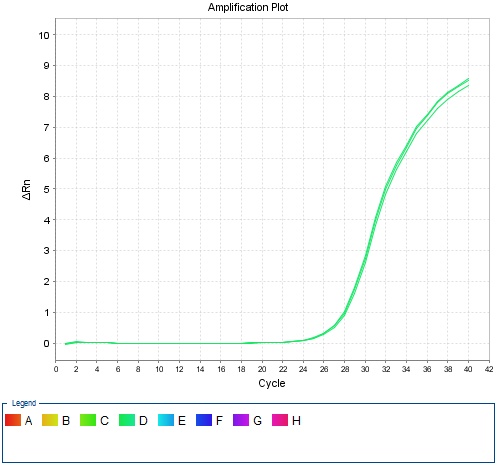


*VmSWEET2.1*
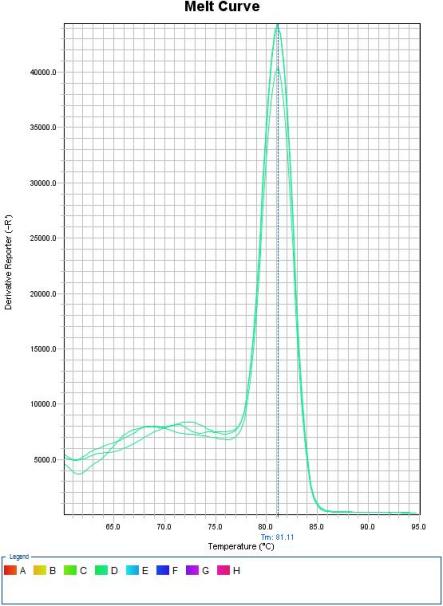

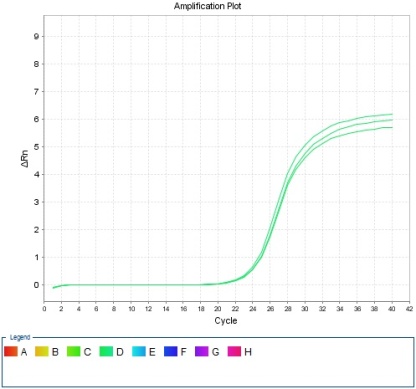


*VmSWEET13
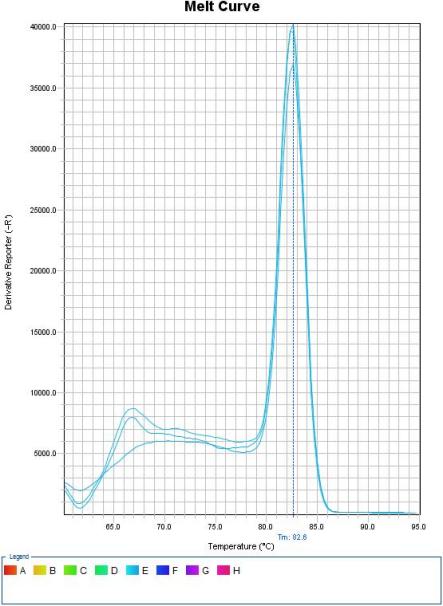

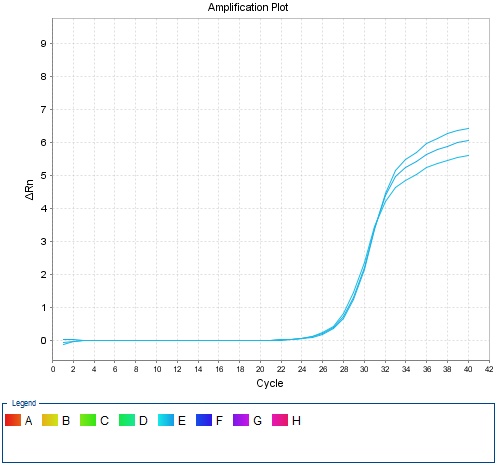
*

*VmSWEET4*
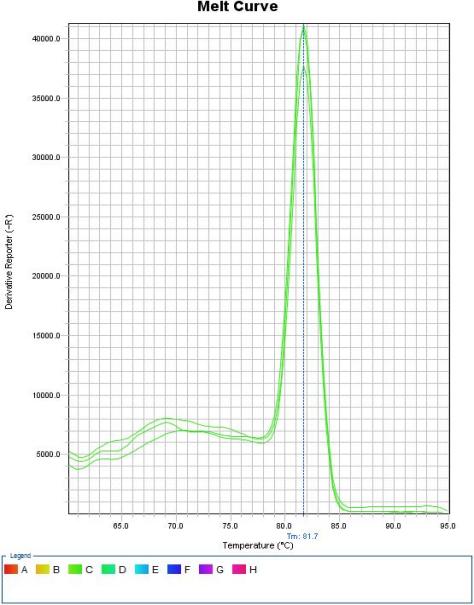

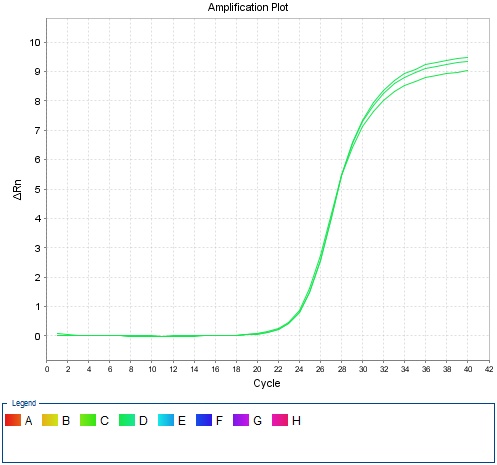


*VmSWEET14*
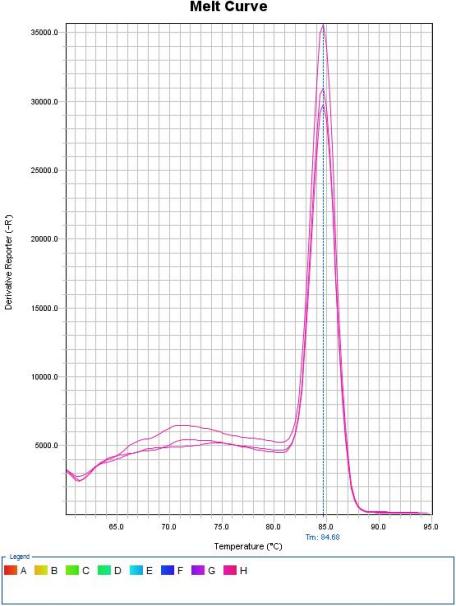

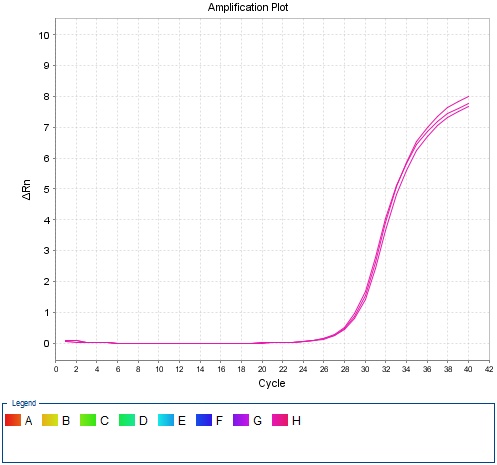


*VmSWEET5*
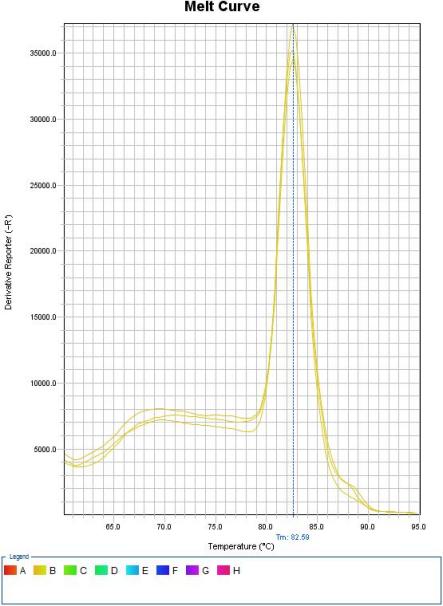

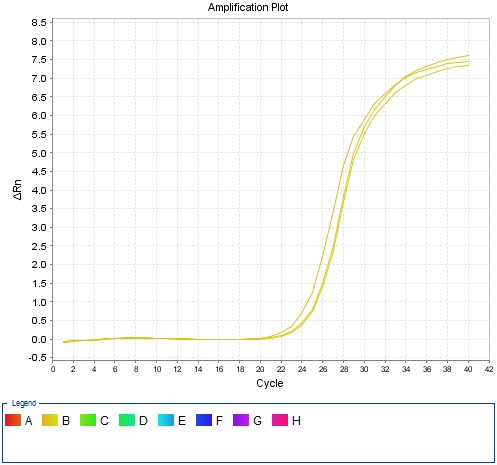


*VmSWEET2.2*
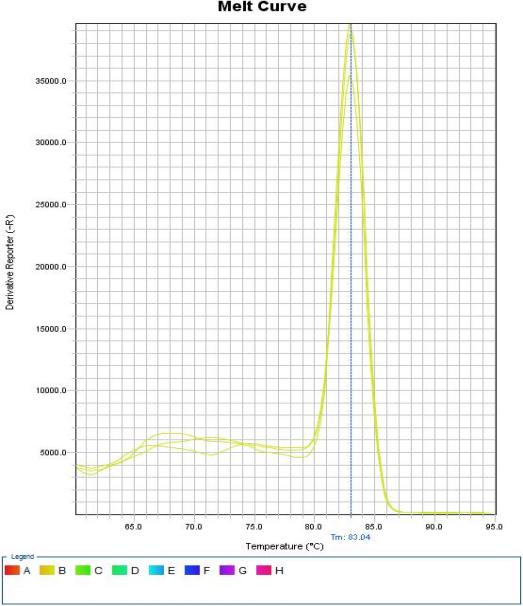

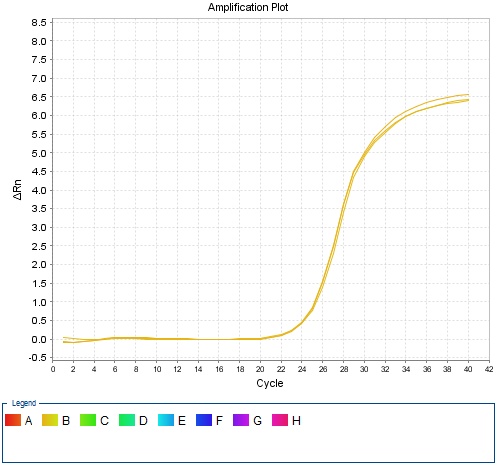


**7.2 Calibration curves and PCR efficiency calculated from slope**

To verify the primer amplification efficiency and create standard curves, a concentration gradient was prepared by serially diluting the cDNA templates with 5-fold dilution to give 1, 1/5, 1/25, 1/125, and 1/625 times dilutions.A standard curve was drawn from the obtained Ct values to obtain the slope k and linear correlation coefficient (regression coefficient) R2. The amplification efficiency E (E = 10^(−1/k)^ − 1) was calculated using the obtained slope k.

**
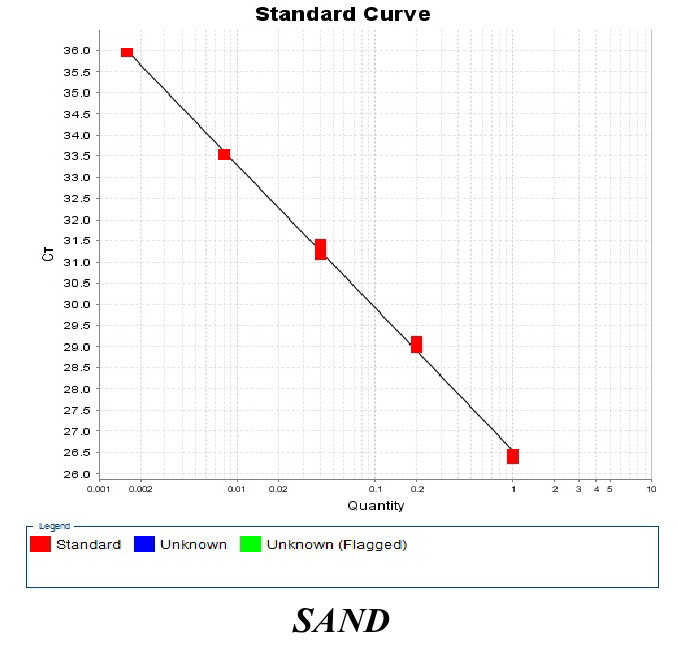
**

K=3.47 PCR efficiency*E*=97.4%

R^2^=0.998

**7.3 Results for NTCs**

*VmSAND*
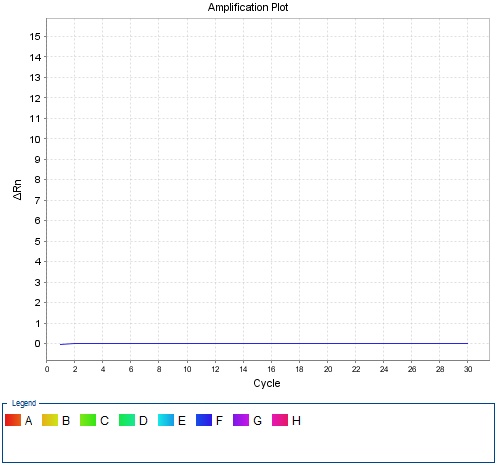

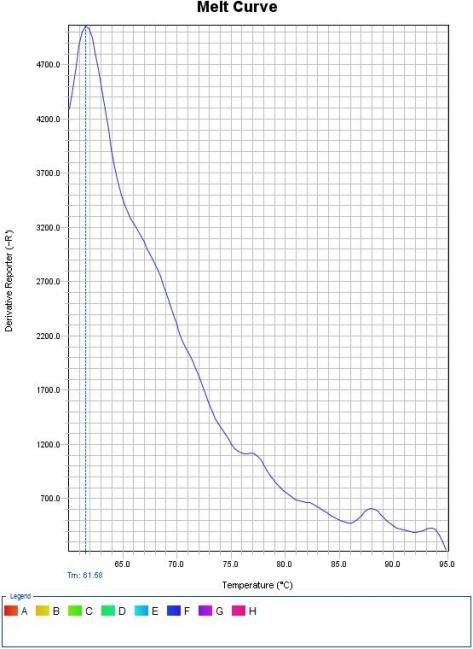


*VmSWEET10.1*
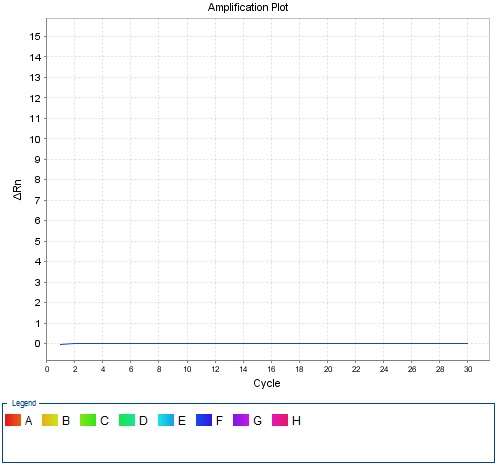

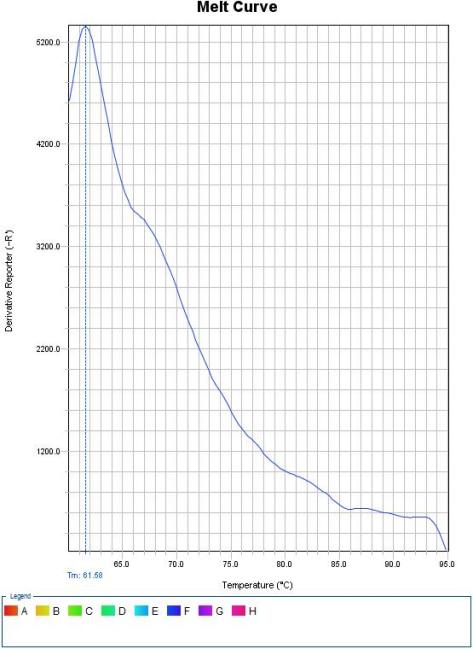


*VmSWEET10.2*
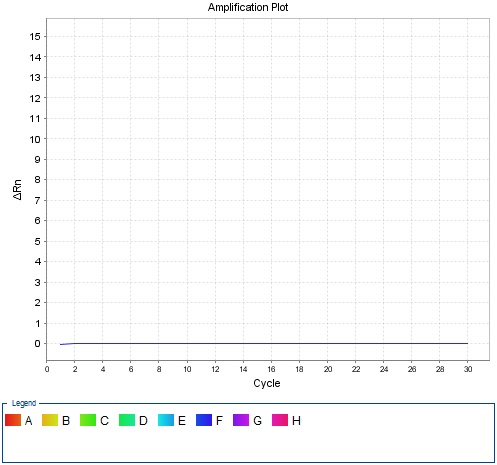

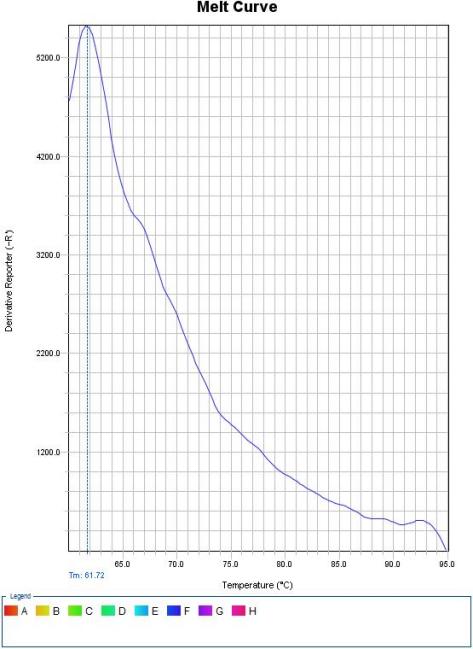


*VmSWEET12*
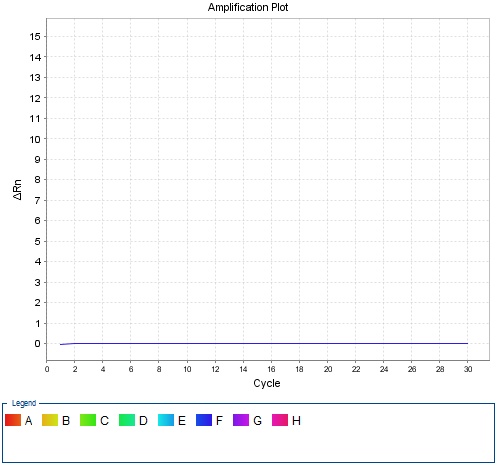

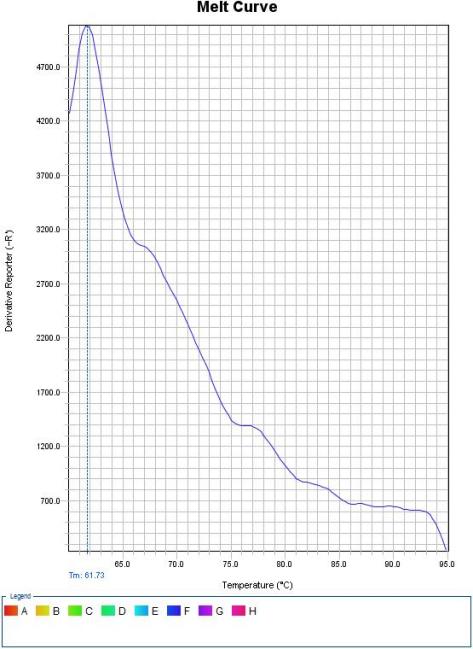


*VmSWEET1.1*
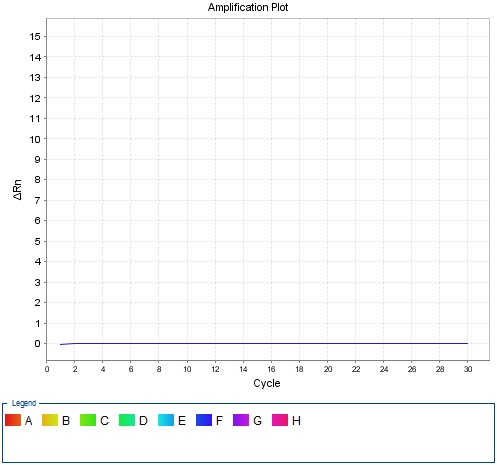

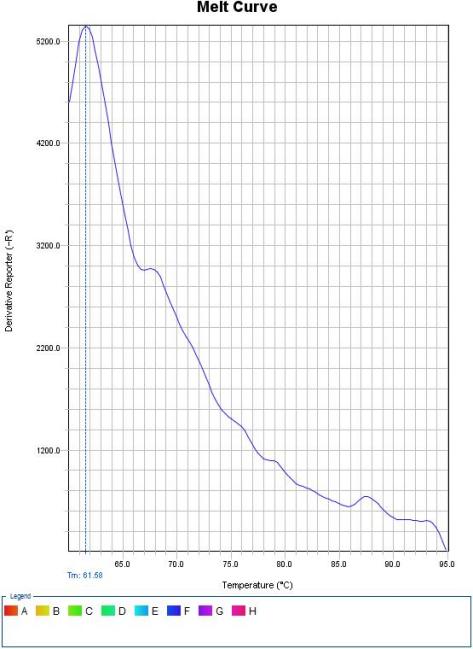


*VmSWEET16*
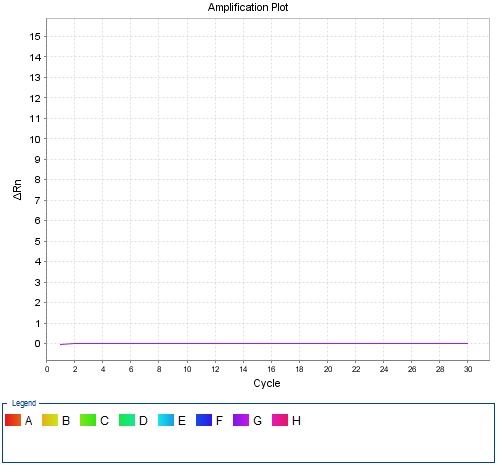

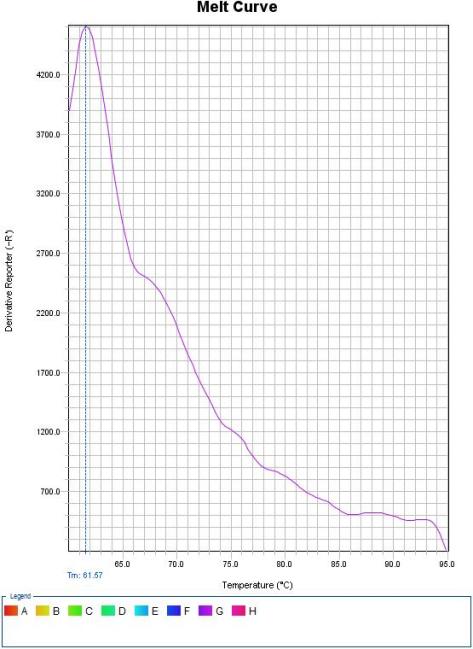


*VmSWEET1.2
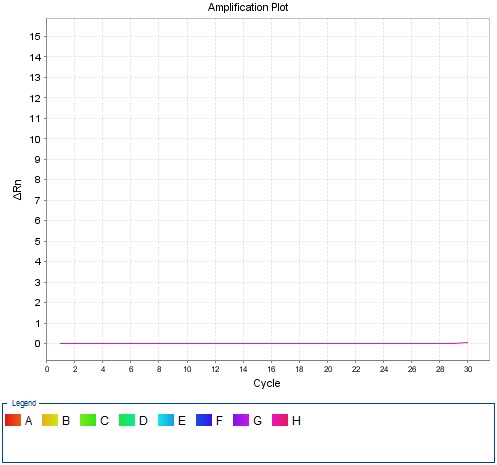

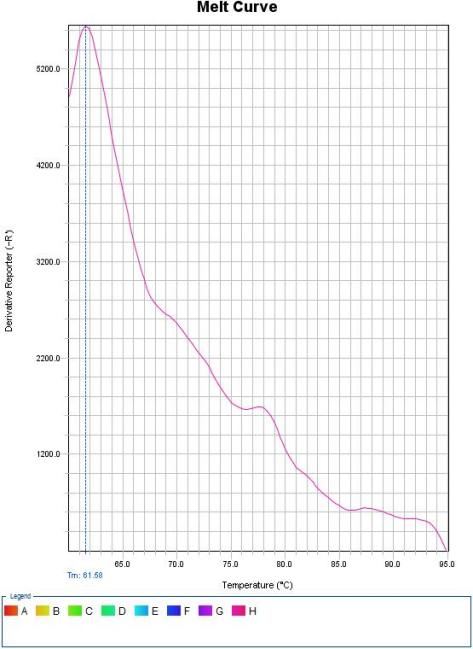
*

*VmSWEET3*
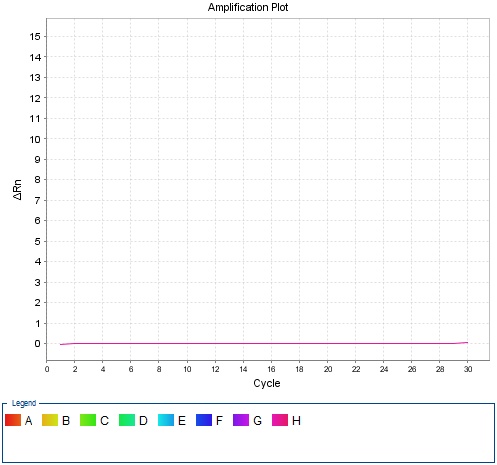

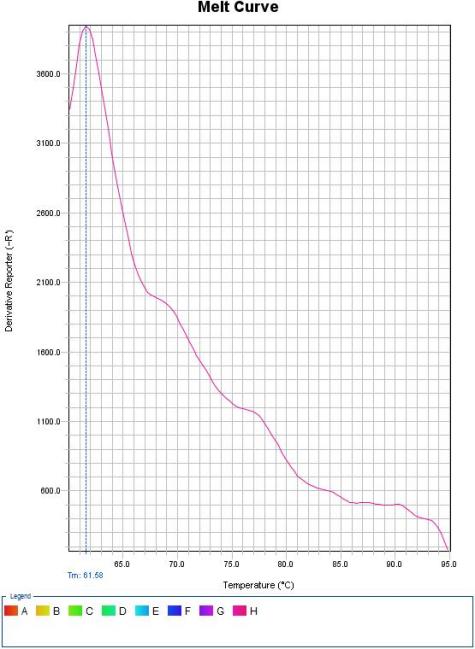


*VmSWEET2.1
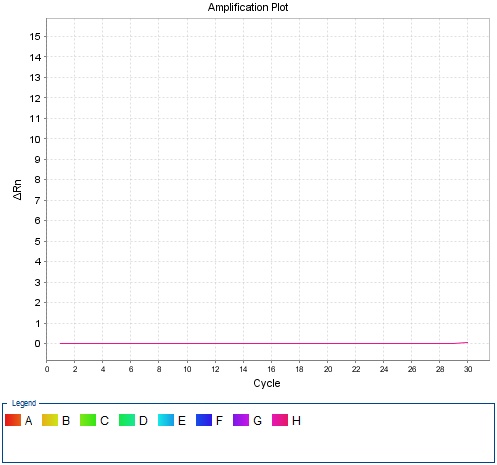
****
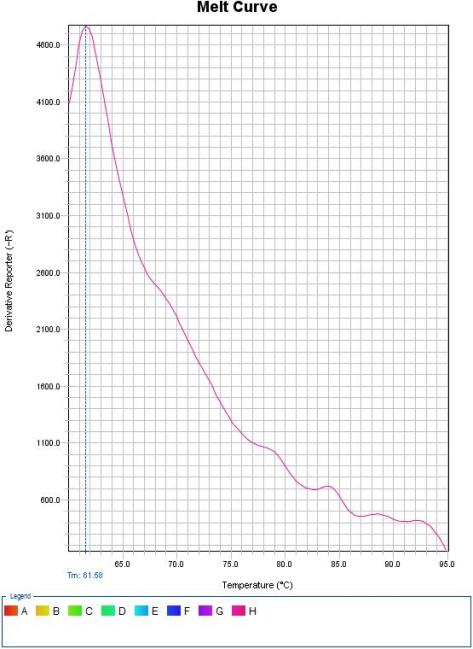
***

*VmSWEET13
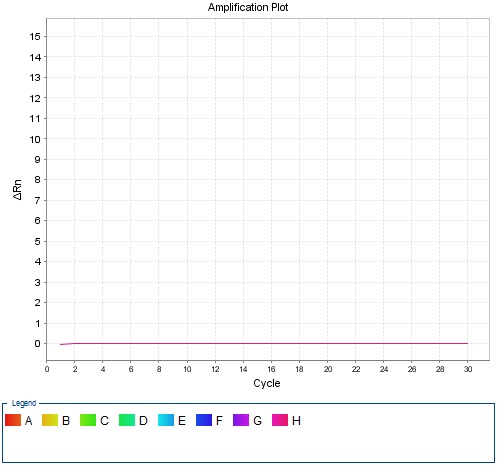

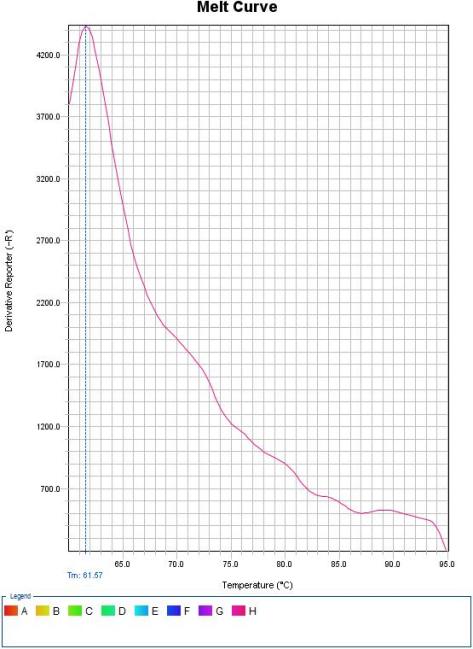
VmSWEET4
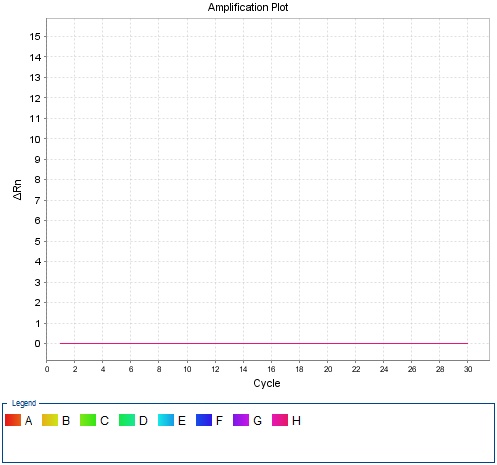

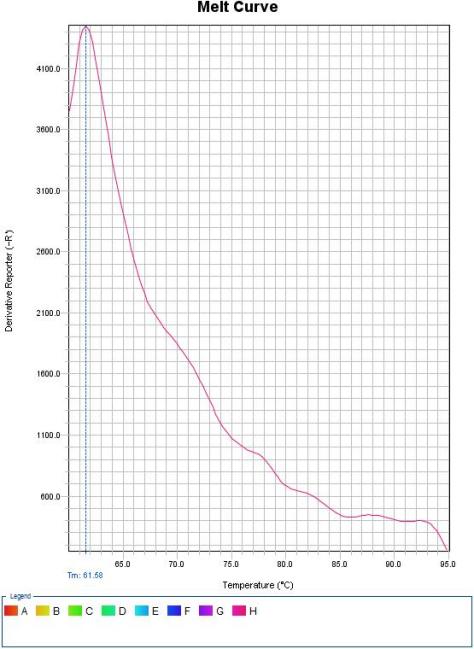
VmSWEET14
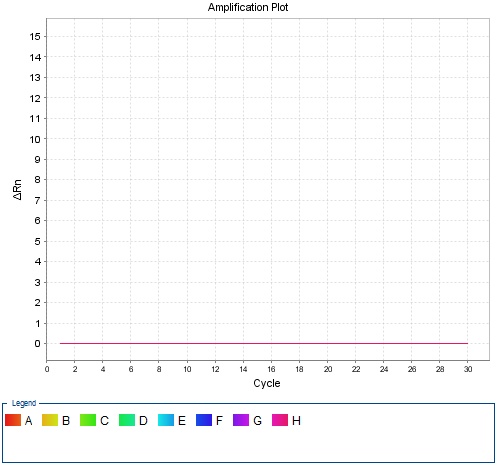

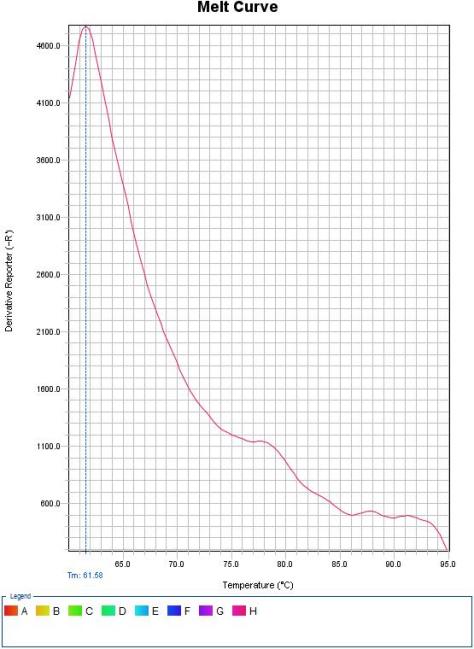
*

*VmSWEET5
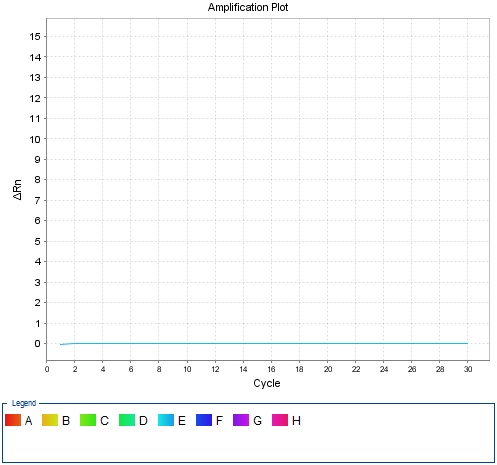

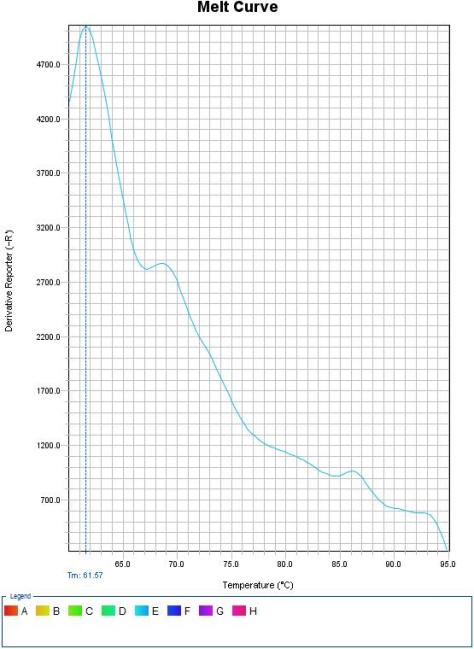
*

*VmSWEET2.2
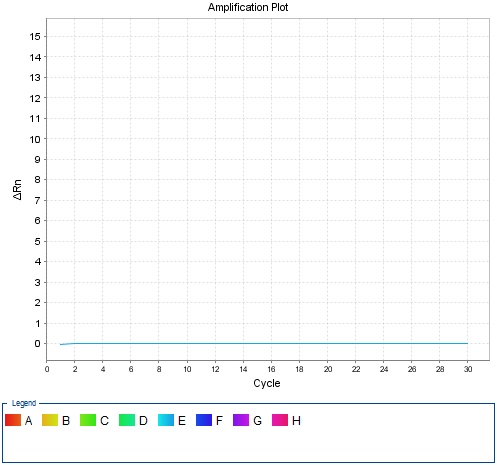

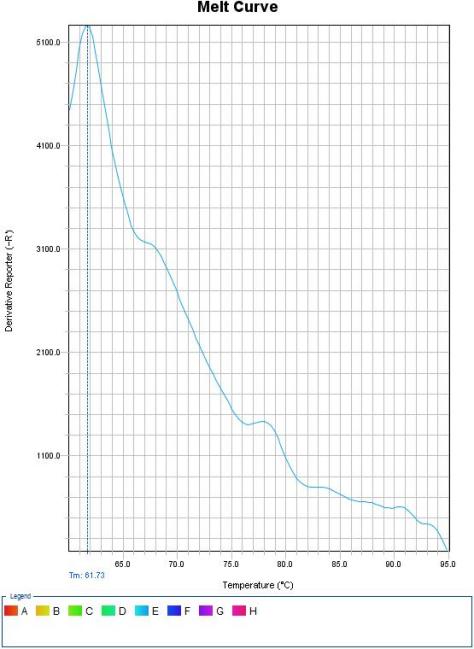
*

**8 Data analysis**

**8.1 qPCR analysis program (source, version)**

StepOne Software v2.3

**8.1 Cq method determination**

The Cqvalue range is between 15-35.

**8.1 Outlier identification and disposition**

The Cq exceeding 35 indicates that it has not been amplified.If the peak starts before 15, it may caused by the high template concentration, we should changed the template concentration.

**8.2 Statistical methods for results significance**

One-WayANOVA（LSD）

**8.3 Software (source, version)**

SPSSv28.0.1.1

**8.4 Justification of number and choice of reference genes**

In previous research, we selected ten candidate reference gene families (*ACTIN*, *CYP2*, *EF-1α*, *F-box*, *GAPDH*, *18s rRNA, TUBB*, *SAND*, *PP2A* and *RH8*) and used three statistical software tools-geNorm, NormFinder and BestKeeper-to evaluate their expression stability under the influence of different experimental factors. The results showed that protein phosphatase 2A regulatory subunit (PP2A) or RNA helicase-like 8 (RH 8) was the best choice for an internal reference gene when analyzing different cranberry cultivars. In two sample sets comprising different cranberry organs and three abiotic stress treatments, sand family protein (SAND) was the best choice as a reference gene. The results published on “Selection of reference genes for normalization of cranberry (Vaccinium macrocarpon Ait.) gene expression under different experimental conditions”. PLoS One. 2019 Nov 12;14(11):e0224798. doi: 10.1371/journal.pone.0224798. PMID: 31715627; PMCID: PMC6850891.

**8.5 Description of normalization method**

Relative quantitative analysis of 13 target genes in different cranberry tissues and fruit development stages were calculated using the 2^–ΔCt^ method, and column charts were obtained by SigamaPlot 10.0. Expression profiles of *VmSWEETs* under abiotic stress were calculated using the using the 2^–ΔΔCt^ method, then the expression level was log_2_ transformed and normalized to obtain a heatmap by TBtools.
