# Supplementary material for: Genome-wide identification and expression analyses of SWEET gene family reveal potential roles in plant development, fruit ripening and abiotic stress responses in cranberry (Vaccinium macrocarpon Ait)
Source: PeerJ. 2024 Sep 19;12:e17974. doi: 10.7717/peerj.17974 (PMC11416763; doi:10.7717/peerj.17974)

Supplementary file 7

Multiple sequence alignment of *OsSWEET2b, AtSWEET1* and *VmSWEETs*. The sequences contained in the black boxes are conserved domains of *VmSWEETs* members. The position of the S, T, and Y predicted to be the phosphorylation sites are indicated by the red triangles. A conserved asparagine pair Asparagine (N77 and N197) in *OsSWEET2b* and a serine (S54) as well as tryptophan (W176) in *AtSWEET1*, which surround the binding pocket associated with the transportation capacity, are indicated by the red arrows.


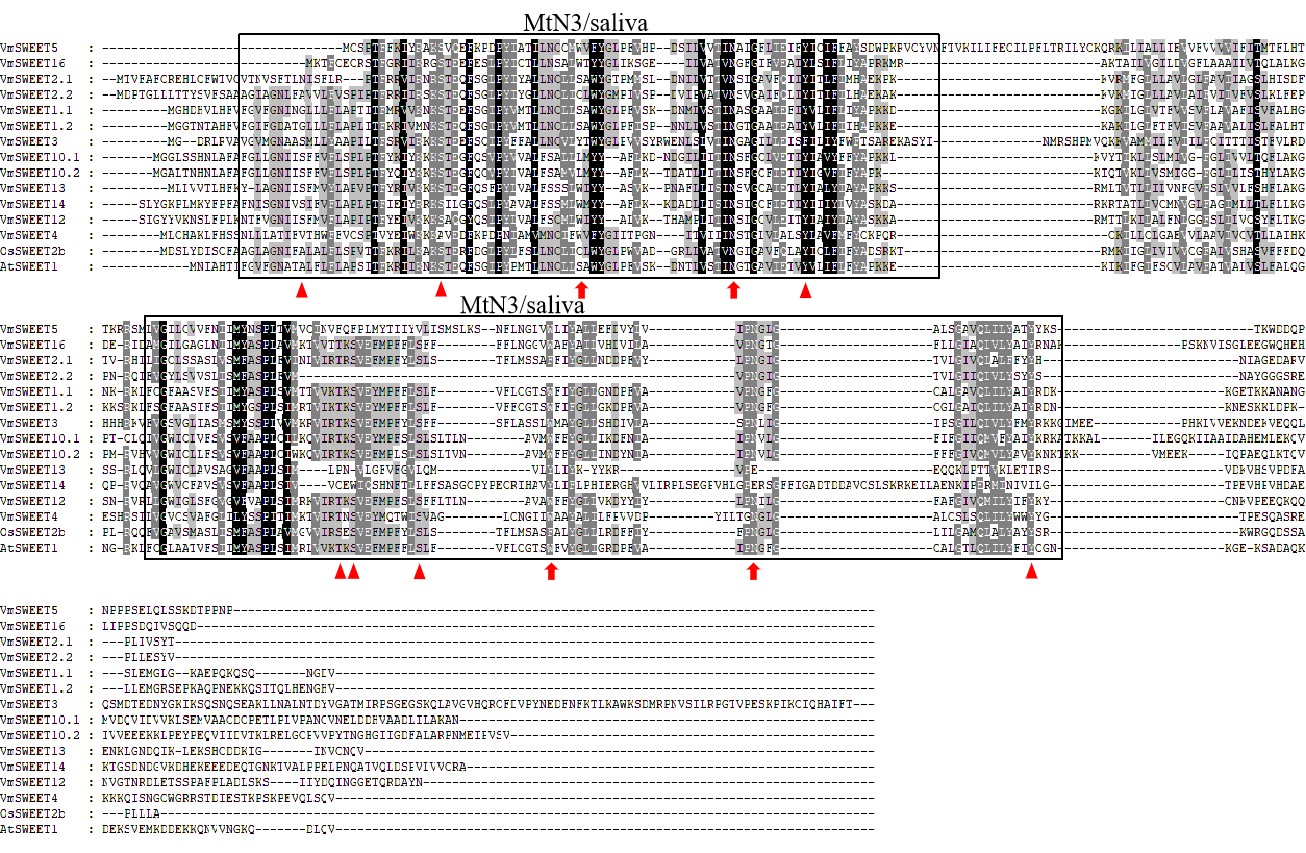

Supplement: Supplemental Information 7 — The sequences contained in the black boxes are conserved domains of VmSWEETs members. The position of the S, T, and Y predicted to be the phosphorylation sites are indicated by the red triangles. A conserved asparagine pair Asparagine (N77 and N197) in OsSWEET2b and a serine (S54) as well as tryptophan (W176) in AtSWEET1 , which surround the binding pocket associated with the transportation capacity, are indicated by the red arrows. [file peerj-12-17974-s007.doc]
