# Supplementary material for: Genome-wide identification and expression analyses of SWEET gene family reveal potential roles in plant development, fruit ripening and abiotic stress responses in cranberry (Vaccinium macrocarpon Ait)
Source: PeerJ. 2024 Sep 19;12:e17974. doi: 10.7717/peerj.17974 (PMC11416763; doi:10.7717/peerj.17974)

Supplementary file 8

The different conserved motifs in cranberry. The letter size of the amino acid represents the frequency of the corresponding nucleotide.


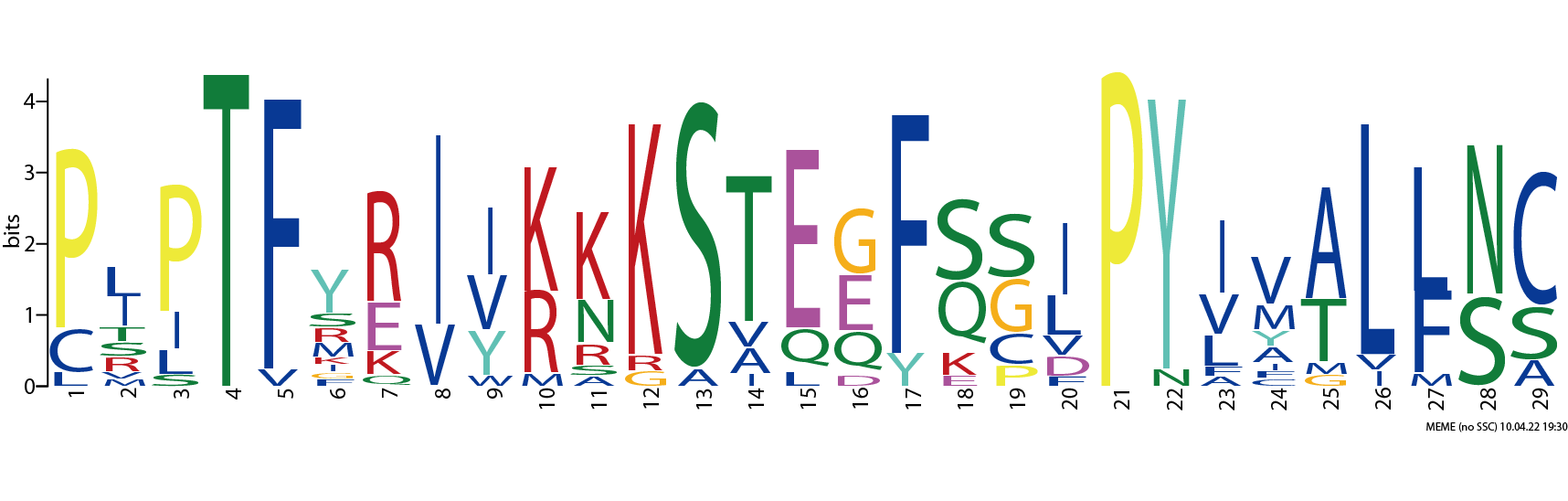


Motif1


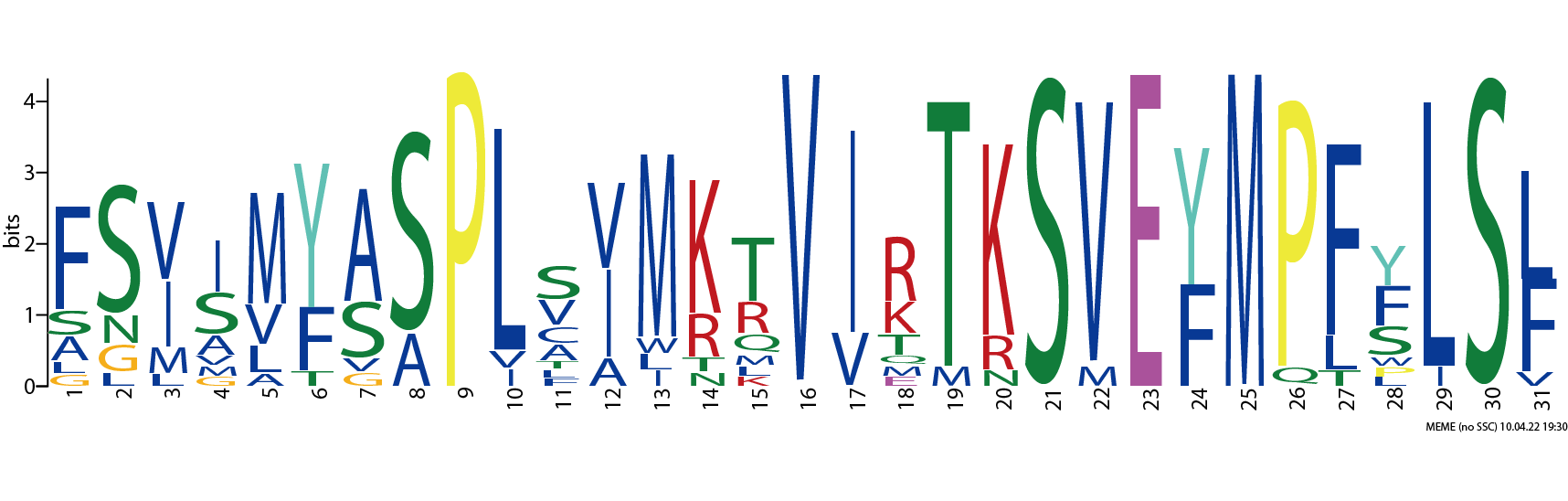


Motif2


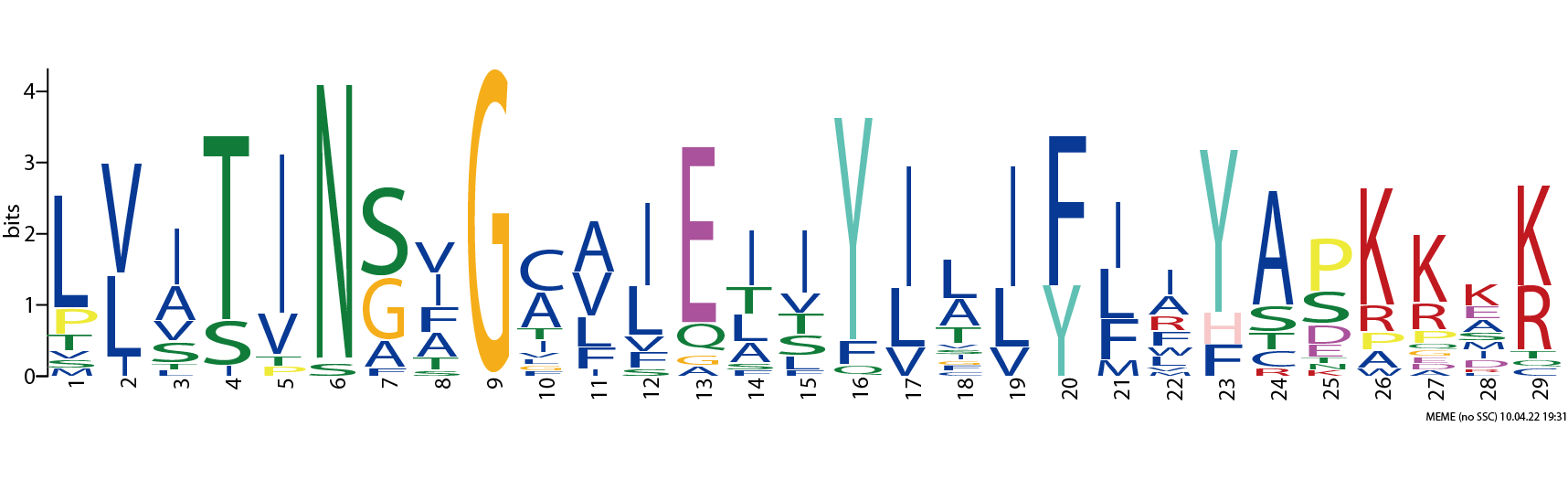


Motif3

Motif4


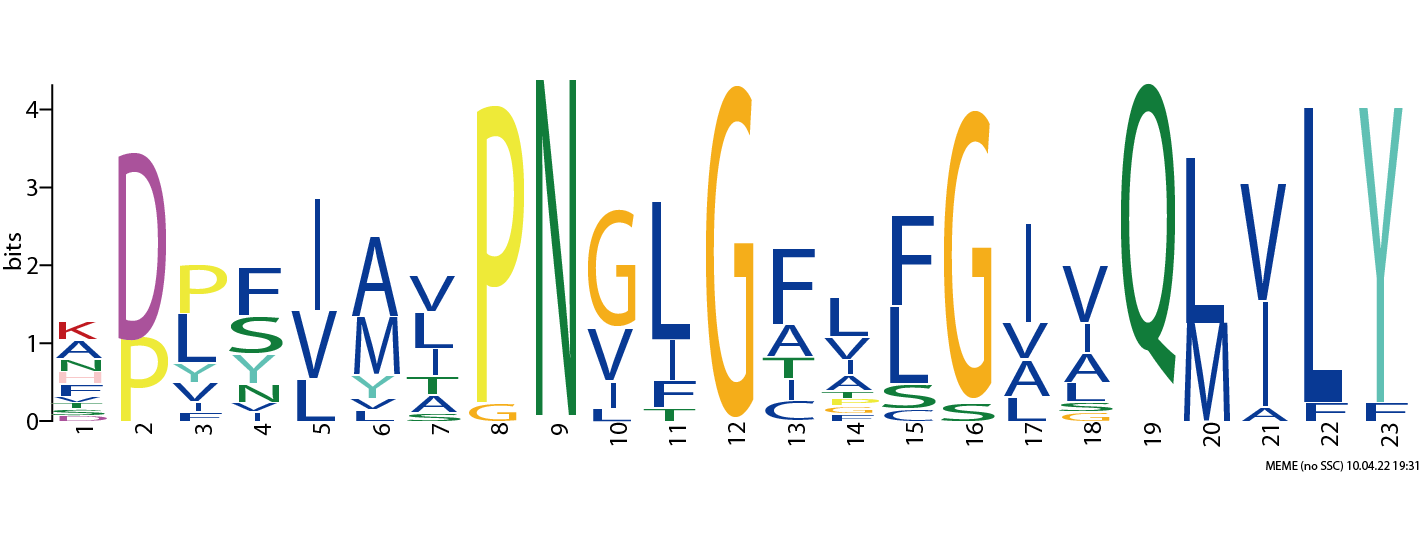


Motif5


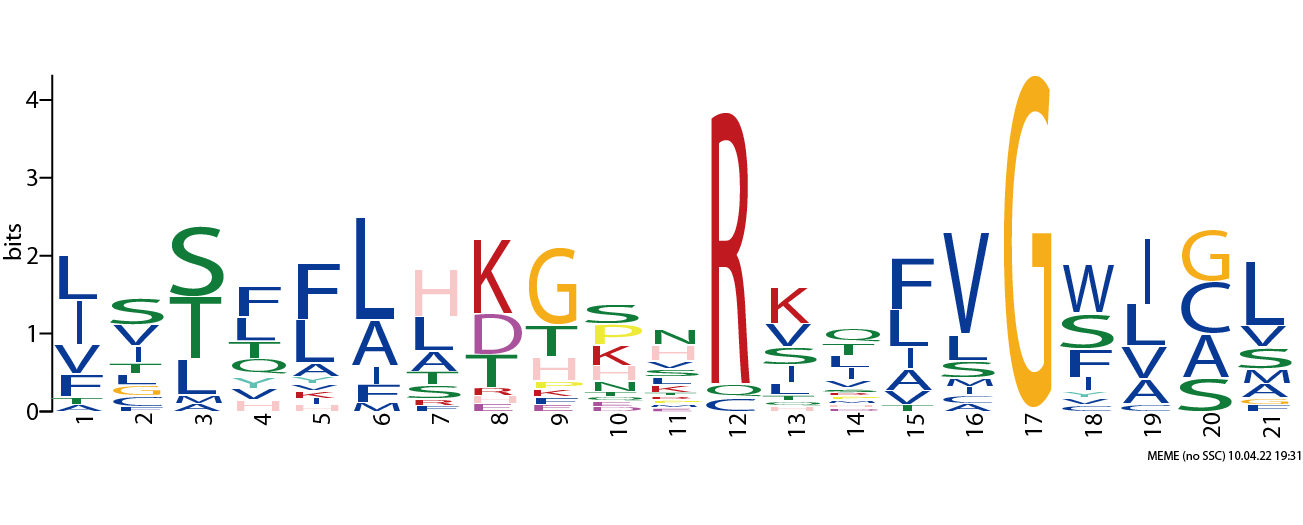


Motif9


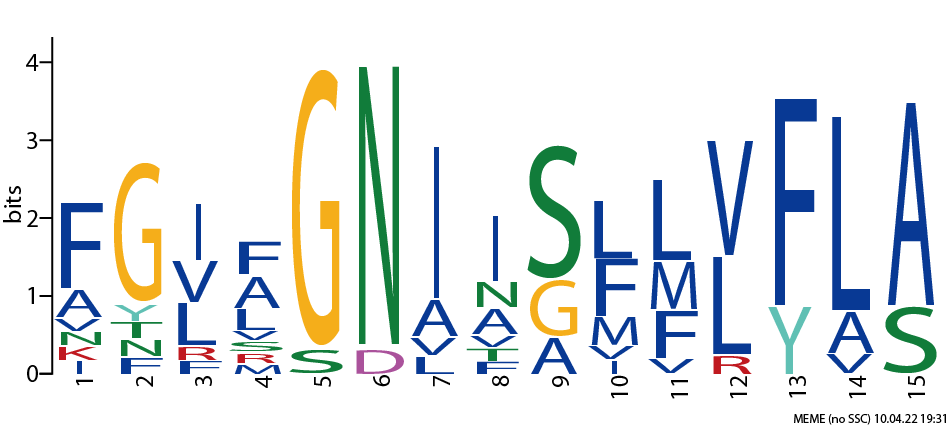


Motif8


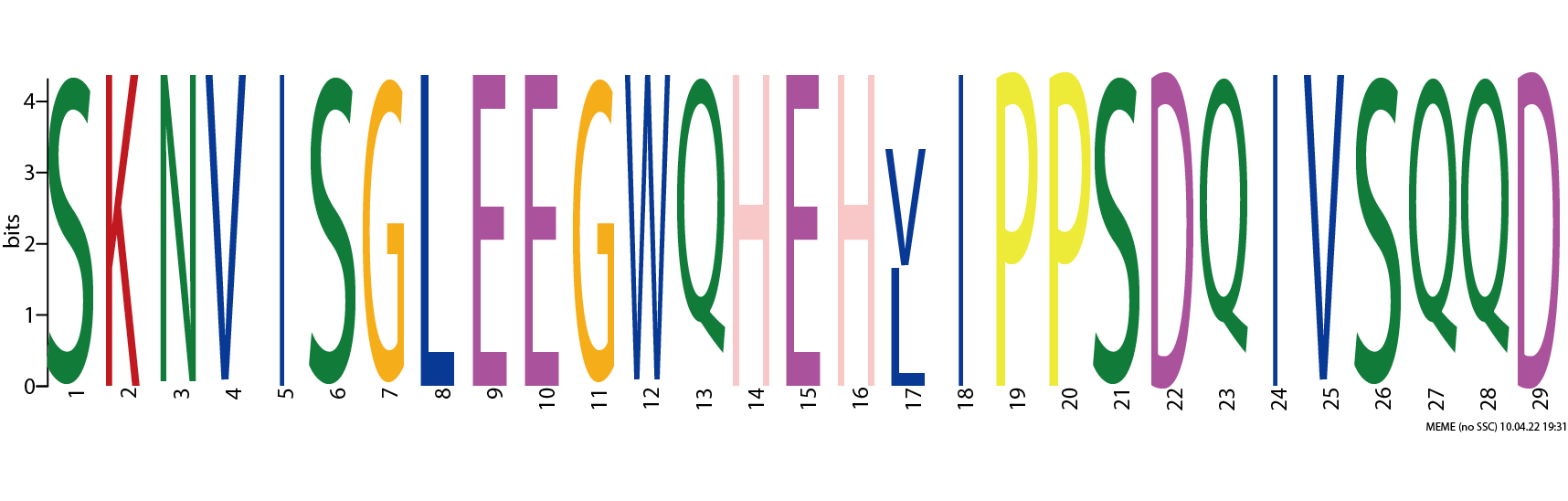


Motif7


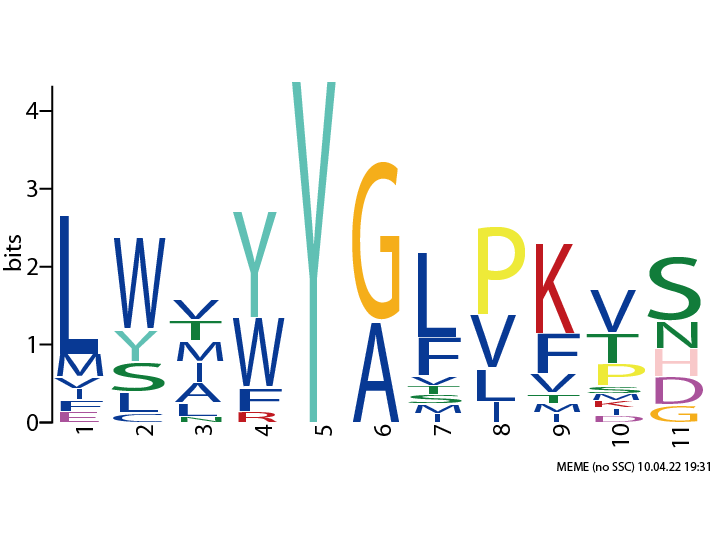


Motif6


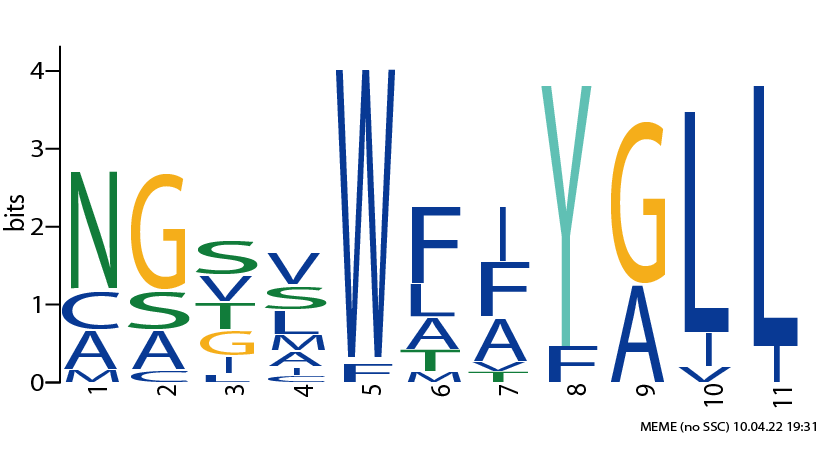

Supplement: Supplemental Information 8 — The letter size of the amino acid represents the frequency of the corresponding nucleotide. [file peerj-12-17974-s008.docx]
