# Supplementary figures and images for: Genome-wide identification and expression analyses of SWEET gene family reveal potential roles in plant development, fruit ripening and abiotic stress responses in cranberry (Vaccinium macrocarpon Ait)
Source: PeerJ. 2024 Sep 19;12:e17974. doi: 10.7717/peerj.17974 (PMC11416763; doi:10.7717/peerj.17974)

Supplementary file 9

Gene structure of cranberry *SWEET* gene family


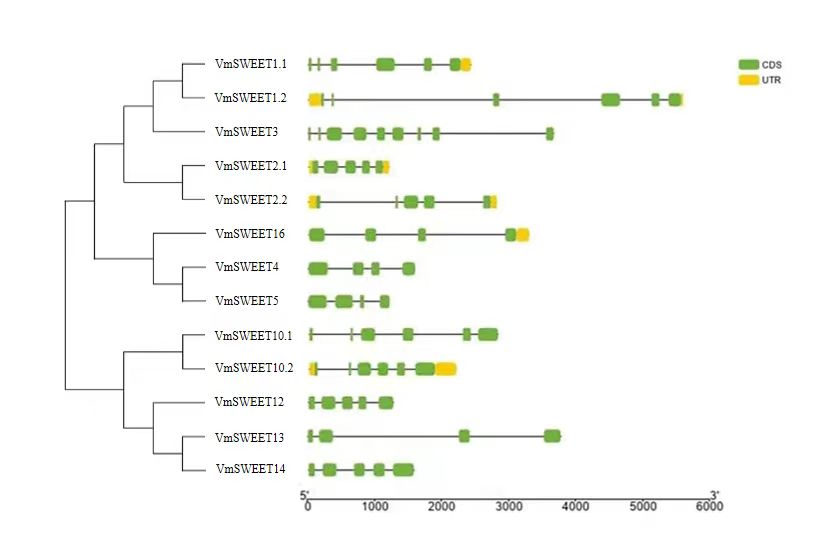

Supplement: Supplemental Information 9 [file peerj-12-17974-s009.doc]

Supplementary file 10

Chromosome mapping of *SWEET* genes in cranberry


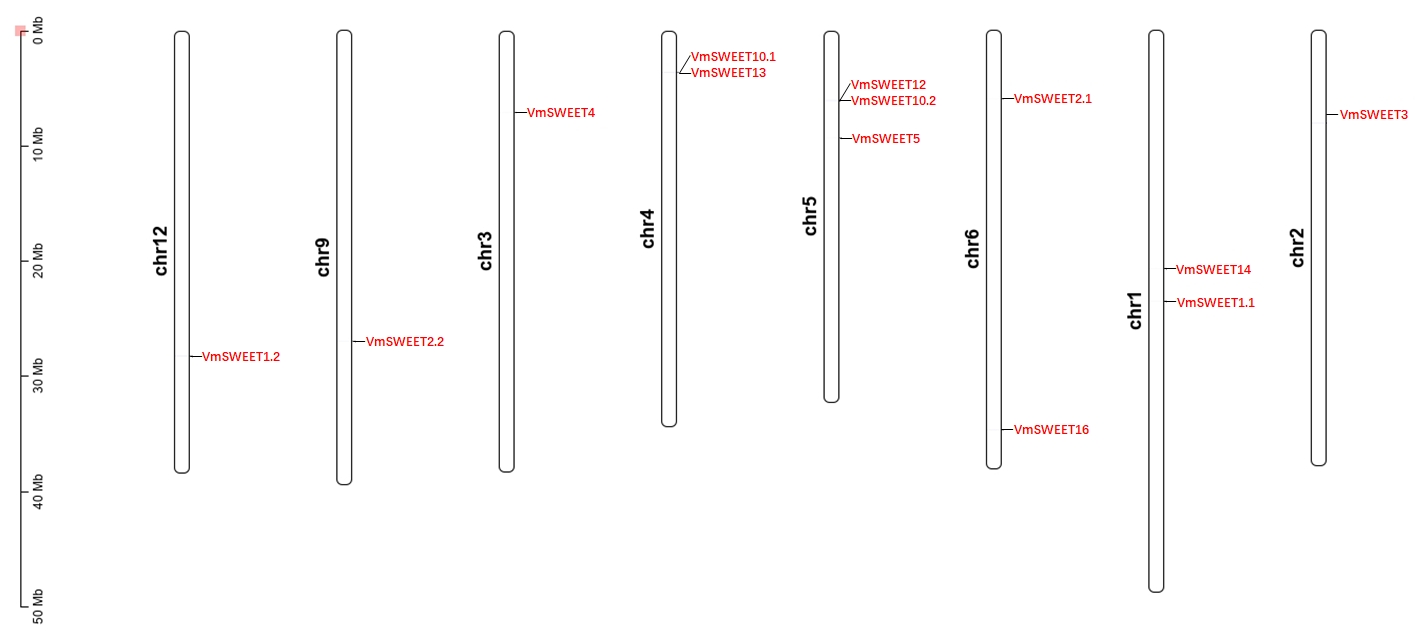

Supplement: Supplemental Information 10 [file peerj-12-17974-s010.doc]
